# The utility of pathway selective estrogen receptor ligands that inhibit nuclear factor-κB transcriptional activity in models of rheumatoid arthritis

**DOI:** 10.1186/ar1692

**Published:** 2005-02-21

**Authors:** James C Keith, Leo M Albert, Yelena Leathurby, Max Follettie, Lili Wang, Lisa Borges-Marcucci, Christopher C Chadwick, Robert J Steffan, Douglas C Harnish

**Affiliations:** 1 Cardiovascular and Metabolic Disease Research, Wyeth Research, Cambridge, MA, USA; 2 Department Biological Technologies, Cambridge, MA, USA; 3 Cardiovascular and Metabolic Disease Research, Collegeville, PA, USA; 4 Women's Health Research Institute, Collegeville, PA, USA; 5 Chemical and Screening Sciences, Collegeville, PA, USA

## Abstract

Rheumatoid arthritis (RA) is a chronic inflammatory disease that produces synovial proliferation and joint erosions. The pathologic lesions of RA are driven through the production of inflammatory mediators in the synovium mediated, in part, by the transcription factor NF-κB. We have identified a non-steroidal estrogen receptor ligand, WAY-169916, that selectively inhibits NF-κB transcriptional activity but is devoid of conventional estrogenic activity. The activity of WAY-169916 was monitored in two models of arthritis, the HLA-B27 transgenic rat and the Lewis rat adjuvant-induced model, after daily oral administration. In both models, a near complete reversal in hindpaw scores was observed as well as marked improvements in the histological scores. In the Lewis rat adjuvant model, WAY-169916 markedly suppresses the adjuvant induction of three serum acute phase proteins: haptoglobin, α1-acid glycoprotein (α1-AGP), and C-reactive protein (CRP). Gene expression experiments also demonstrate a global suppression of adjuvant-induced gene expression in the spleen, liver, and popliteal lymph nodes. Finally, WAY-169916 was effective in suppressing tumor necrosis factor-α-mediated inflammatory gene expression in fibroblast-like synoviocytes isolated from patients with RA. Together, these data suggest the utility of WAY-169916, and other compounds in its class, in treating RA through global suppression of inflammation via selective blockade of NF-κB transcriptional activity.

## Introduction

Rheumatoid arthritis (RA) is a chronic, debilitating condition affecting 0.5 to 1% of the world's population. The major goals of treatment of RA are to reduce pain and discomfort, to prevent deformities and loss of joint function, and to maintain a productive and active lifestyle. RA is characterized by chronic joint inflammation mediated by inflammatory cell infiltration into synovial tissues as well as joint destruction through the overexpression of matrix metalloproteinase (MMP) in articular synoviocytes and chondrocytes. The pathologic lesions of RA are driven, in part, by the production of inflammatory mediators in synoviocytes and macrophages, probably involving the transcription factor NF-κB. Because NF-κB is localized in the nuclei of synovial cells in patients with RA [1,2] and the inducers and targets of NF-κB almost perfectly match the list of pivotal mediators increased in RA [3], an important role for activated NF-κB in human RA is likely.

NF-κB is a dimeric transcription factor composed of homodimeric and heterodimeric complexes of the Rel family of proteins, p65 (Rel A), p50/105, c-Rel, p52/100, and Rel B. Binding of cytoplasmic inhibitory protein-κB (IκB) to NF-κB masks the NF-κB nuclear localization signal and sequesters NF-κB in a non-activated form in the cytoplasm. Cell activation by a variety of extracellular signals such as oxidative stress, cytokines, and lipopolysaccharide induces a cascade of events that leads to the degradation of IκB; activated NF-κB then translocates to the nucleus, where it binds to DNA elements in the promoters of several proinflammatory gene families [4].

Activation of NF-κB has been observed in synovial cells from patients with RA [5] and results in the induction of proinflammatory genes such as tumor necrosis factor-α (TNF-α), IL-1β, IL-6, MMP-1, and MMP-3 in *ex vivo *synovial membrane cultures [6]. Moreover, NF-κB activation might also be a pivotal factor protecting cells from apoptosis, thus contributing to synovial hyperplasia (reviewed in [7]). Inactivation of NF-κB in transgenic mice expressing a 'super-repressor' IκBα or in rel- /- and *nfkb*1- /- knockout mice rendered the animals refractory to development of collagen-induced arthritis [8,9]. In another study performed in the rat adjuvant-induced arthritis model, intra-articular injection of an adenoviral construct encoding a dominant-negative from of IκB kinase-2 significantly ameliorated the severity of the adjuvant arthritis and was correlated with a decrease in NF-κB DNA binding in the nucleus of synovial cells [10]. Because NF-κB is involved in normal immune and homeostatic processes, its prolonged inhibition might be harmful. Therefore, more indirect methods of targeting NF-κB might provide a safer pharmacological profile.

In tissues that express estrogen receptor (ER), 17β-estradiol inhibits NF-κB-driven transcription through multiple mechanisms that might include direct protein–protein interactions [11,12], inhibition of NF-κB binding to DNA [13,14], induction of IκB expression [15], or coactivator sharing [16,17]. Two nuclear estrogen receptors have been identified (ERα and ERβ). Both receptors are widely distributed throughout numerous organs [18] and are present in T cells, monocytes, dendritic cells, synovial macrophages, articular chondrocytes, and proliferating fibroblasts present in the RA joint [19-22]. These two receptors have a nearly identical DNA-binding domain, both activate transcription through binding to identical ER response elements [23,24], and both can antagonize NF-κB transcriptional activity [25,26]. Taken together, these findings identify RA as a disease amenable to treatment with ER-selective NF-κB inhibitors.

The selective inflammatory modulator WAY-169916 is a non-steroidal ER-dependent inhibitor of NF-κB transcriptional activity. Although it inhibits the expression of a range of inflammatory proteins, including cytokines, chemokines, and cell adhesion molecules that are expressed after activation of NF-κB, WAY-169916 lacks estrogenic activity such as the stimulation of uterine proliferation [27]. Here we demonstrate that WAY-169916 is efficacious in two models of arthritis, the HLA-B27 transgenic rat and a Lewis rat model of adjuvant-induced arthritis. The activity of WAY-169916 is related to its ability to suppress inflammatory processes globally, as demonstrated by the decrease in serum acute-phase protein levels of haptoglobin, α1-acid glycoprotein (α1-AGP), and C-reactive protein (CRP) as well as the inhibition of adjuvant-induced gene expression in the spleen, liver, and popliteal lymph nodes in the rat adjuvant arthritis model. Moreover, WAY-169916 was also active in suppressing cytokine and adhesion molecule expression in fibroblast-like synoviocytes (FLS) isolated from patients with RA. Taken together, these data suggest the potential utility of the pathway-selective ER ligand WAY-169916 and other compounds in its class in the treatment of RA.

## Materials and Methods

### Animals

Male HLA-B27 transgenic rats were obtained from Taconic; the Lewis rats were purchased from Charles River Laboratories. The rats were housed in accordance with standard operating procedures and were provided with food and water *ad libitum*. All experiments were approved and performed in accordance with the Wyeth Animal Care and Use Committee standards.

### HLA-B27 transgenic rat model arthritis

HLA-B27 transgenic rats, 26 to 28 weeks old, experiencing maximal clinical signs of arthritis with a score of 12, using a scale of 0 to 3 for swelling and for erythema of the hindpaws (0, normal paw; 1, mild; 2, moderate; 3, severe) were treated with vehicle (2% Tween 80, 0.5% methylcellulose), prednisolone (0.6 mg/kg), or WAY-169916 (10 mg/kg) given orally once daily for 29 days with four rats per group. At necropsy, the tarsal joints were removed and prepared for histological examination. After decalcification, histological sections were stained with hematoxylin and eosin or Safranin O/Fast Green stain. Synovial tissue from tarsal joints was evaluated on the basis of synovial hyperplasia, fibroplasia, inflammatory cell infiltration, and pannus formation [28].

Articular cartilage was evaluated with Mankin's histological grading system [29]. The scoring system evaluates the structure of the articular cartilage, ranging from 0 (normal), 1 (surface irregularity), 2 (pannus and surface irregularity), 3 (clefts to transititional zone), 4 (clefts to radial zone), 5 (clefts to the calcified zone), to 6 (complete disorganization; cartilage cells, ranging from 0 (normal), 1 (diffuse hypercellularity), 2 (cloning), to 3 (hypocellularity); Safranin-O staining to assess proteoglycan content, ranging from 0 (normal), 1 (slight reduction), 2 (modest reduction), 3 (severe reduction), to 4 (no staining); and tidemark integrity, ranging from 0 (intact) to 1 (crossed by blood vessels). The scores for eachtarsal joint were tabulated and summed, and amean score was derived for each animal, ranging from 0 to 14. Statistical analysis was performed with Abacus Concepts Super ANOVA (Abacus Concepts, Inc., Berkeley, CA). All parameters of interest were subjected to analysis of variance (ANOVA) with Duncan's new multiple-range post hoc testing between groups. Data are expressed throughout as means ± standard deviation (SD), and differences were deemed significant if *P *< 0.05.

### Rat adjuvant-induced arthritis model

Arthritis was induced in the Lewis rats with complete Freund's adjuvant (CFA) by intradermal injection of 0.1 mg of heat-killed and dried *Mycobacterium tuberculosis *in 0.1 ml of mineral oil, at the base of the tail. Eight days after adjuvant injection, when the rats were experiencing maximal clinical signs of arthritis with a score of 12 using the same hindpaw scoring system described above, treatment began. Male Lewis rats (*n *= 6) received orally delivered vehicle (2.0% Tween 80, 0.5% methylcellulose, 1 ml/kg) or WAY-169916 (5.0, 0.3, or 0.1 mg/kg) once daily for 10 to 14 days, with six rats in each group. The clinical signs of arthritis were monitored daily. At the end of the experiment, terminal blood samples were obtained and the tarsal joints were prepared for histological examination and graded as described above. Statistical analysis was performed with Abacus Concepts Super ANOVA. All parameters of interest were subjected to ANOVA with Duncan's new multiple-range post hoc testing between groups. The serum samples were used to determine the levels of haptoglobin, α1-AGP, and CRP by radial immunodiffusion test kits in accordance with manufacturer's protocol (Life Diagnostics Inc.). The data were analyzed by one-way ANOVA and are expressed as means ± SD, and differences were deemed significant if *P *< 0.05.

Gene expression profiling experiments were conducted with RNA isolated from the spleen, liver, and popliteal lymph nodes, using Affymetrix REA230A oligonucleotide arrays (Affymetrix) in accordance with the manufacturer's recommendations. The arrays were washed and stained with Streptavidin R–phycoerythrin (Molecular Probes) with the use of the GeneChip^® ^Fluidics Station 400, and scanned with a Hewlett Packard GeneArray Scanner in accordance with the manufacturer's instructions. Fluorescent data were collected and converted to gene-specific difference averages with MicroArray Suite 4.0 software. A representative set of genes regulated by WAY-169916 was confirmed by real-time RT–PCR analysis. All mRNA levels were normalized for glyceraldehyde-3-phosphate dehydrogenase expression. The data were analyzed by one-way ANOVA and expressed as means ± SD, and differences were deemed significant if *P *< 0.05.

### NF-κB DNA binding experiments

Mouse splenocytes were prepared by creating single-cell suspensions, with the subsequent removal of red blood cells with Tris-NH_4_Cl solution. After lysis of red blood cells, the cells were cultured in 24-well plates at a concentration of 10^6 ^cells/ml in RPMI-10 (RPMI medium containing 10% heat-inactivated fetal bovine serum, 100 U/ml penicillin, 100 μg/ml streptomycin, 2 mM glutamine, and 50 μM 2-mercaptoethanol; Invitrogen, Carlsbad, CA). The cells were stimulated with concanavalin A (ConA) and co-treated with either WAY-169916 (1 μM) or pyrrolidine dithiocarbamate (PDTC; 100 μM) for 18 hours. Nuclear extract preparation and NF-κB DNA binding experiments were conducted with kits purchased from Active Motif.

### Experiments with FLS

Human FLS isolated from patients with RA were purchased from Cell Applications, Inc. The cells were cultured in synoviocyte growth medium (Cell Applications, Inc.) and seeded at 3 × 10^5 ^cells per well in a 12-well dish. After overnight culture, the cells were pretreated for 1 hour with vehicle, WAY-169916 (1 μM), or PDTC (100 μM), followed by stimulation for 1 hour with TNF-α (100 U/ml). Synoviocyte RNA was isolated after the 1 hour of TNF-α treatment, and gene expression analysis was performed using real-time RT–PCR with an ABI PRISM 7900 Sequence Detection System, in accordance with the manufacturer's protocol (Applied Biosystems). The data were analyzed with Sequence Detector v2.1 software (Applied Biosytems) and normalized to glyceraldehyde-3-phosphate dehydrogenase with the Applied Biosystems primer set. Values are reported as means ± SEM for each group from two experiments, with *n *= 3. The data were analyzed by one-way ANOVA and differences were deemed significant if *P *< 0.05.

## Results

### Activity in the hla-b27 transgenic rat

**Figure 1:**
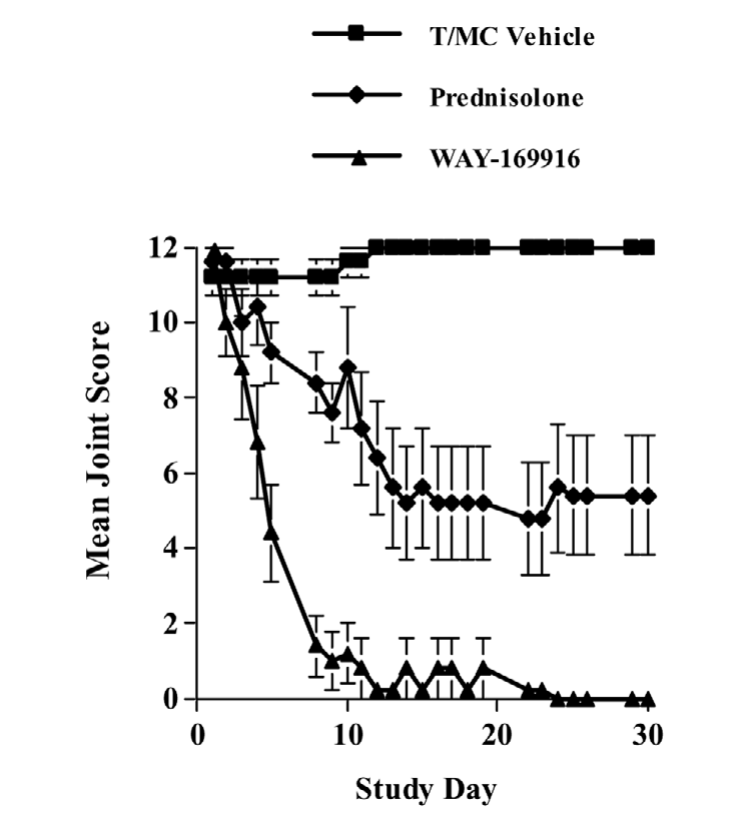
WAY-169916 improves joint scores in HLA-B27 transgenic rat model of arthritis. HLA-B27 transgenic rats, 26 to 28 weeks old, presenting signs of arthritis were treated orally daily with vehicle, prednisolone (0.6 mg/kg), or WAY-169916 (10 mg/kg) for 29 days. Joint scores were assessed by evaluating hindpaws for erythema and swelling (0 to 3 each; maximal score of 12).

The HLA-B27 transgenic rat expresses two human proteins (HLA-B27 and β_2_-microglobulin) that, over time, provoke a misdirected immune response. This model represents a chronic intestinal inflammation with associated arthritis induced by the human class I major histocompatibility allele HLA-B27, which is strongly associated with human disease. Treatment of male HLA-B27 transgenic rats with concentrations of WAY-169916 as low as 0.05 mg/kg rapidly converts the chronic diarrhea that these rats experience to a normal stool [27]. If the disease is allowed to progress, they begin to show symptoms of arthritis. In these settings, treatment of WAY-169916 at a single oral dosage of 10 mg/kg per day restored the clinical joint scores to baseline after 10 days, while a sub-optimal dose of prednisolone (0.6 mg/kg) resulted in a 50% improvement in the joint scores (Fig. 1). Histological scoring of synovitis and cartilage damage in the tarsal joints after 29 days of treatment was also conducted. Treatment with WAY-169916 significantly decreased the synovitis parameters of synovial structure, fibroplasia, inflammatory cell infiltrates, and total synovitis score, and also significantly improved all cartilage parameters monitored (Table 1).

### Activity of WAY-169916 in the Lewis rat adjuvant-induced arthritis model

**Figure 2:**
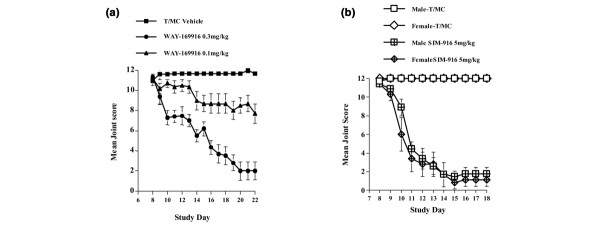
WAY-169916 improves joint scores in a dose-dependent fashion in rat adjuvant-induced arthritis model. **(a) **Male Lewis rats were injected with complete Freund's adjuvant on day 1 and maximal inflammation was allowed to develop. Beginning on day 8 and continuing until day 22, rats were treated daily with oral vehicle or WAY-169916 (0.3 and 0.1 mg/kg). Joint scores were assessed by evaluating hindpaws for erythema and swelling (0 to 3 each; maximal score of 12). **(b) **WAY-169916 improves joint scores in rat adjuvant-induced arthritis model in both males and females. Experiments were performed as in (a) except that WAY-169916 was dosed daily at 5 mg/kg orally in both intact male and intact female rats.

WAY-169916 was then given a more thorough evaluation in the male Lewis rat adjuvant-induced arthritis model. The disease in this model is a migratory polyarthritis affecting primarily the tarsal, metatarsal, and interphalangeal joints. The hallmarks of the model include polyarticular inflammation, marked bone resorption, and periosteal bone proliferation. When immunized with CFA, the joints of Lewis rats swell markedly over a period of 8 days. After maximal swelling had occurred, rats received an oral daily dose of WAY-169916, making this a therapeutic dosing regimen. Joint swelling was rapidly and markedly reduced in rats treated with WAY-169916. Full efficacy was seen with oral doses of 0.3 mg/kg or higher (Fig. 2) but efficacy was decreased at a dose of 0.1 mg/kg. However, both doses were effective at reversing tarsal joint destruction as assessed by synovitis and cartilage (Mankin) scores (Table 2). Incremental improvements in the histology scores were observed with higher doses of WAY-169916 (data not shown), suggesting that continued improvements in joint lesions might occur with a longer duration of treatment or with higher dosages.

Because both the HLA-B27 transgenic rat and Lewis rat studies used males, the efficacy of WAY-169916 (5 mg/kg) was compared in intact male and female Lewis rats with the same experimental design as described above. The joint (Fig. 2b) and histology scores (not shown) for the two sexes were equivalent; it therefore does not seem that the utility of WAY-169916 is restricted by gender.

### Mechanism of action of WAY-169916

**Figure 3:**
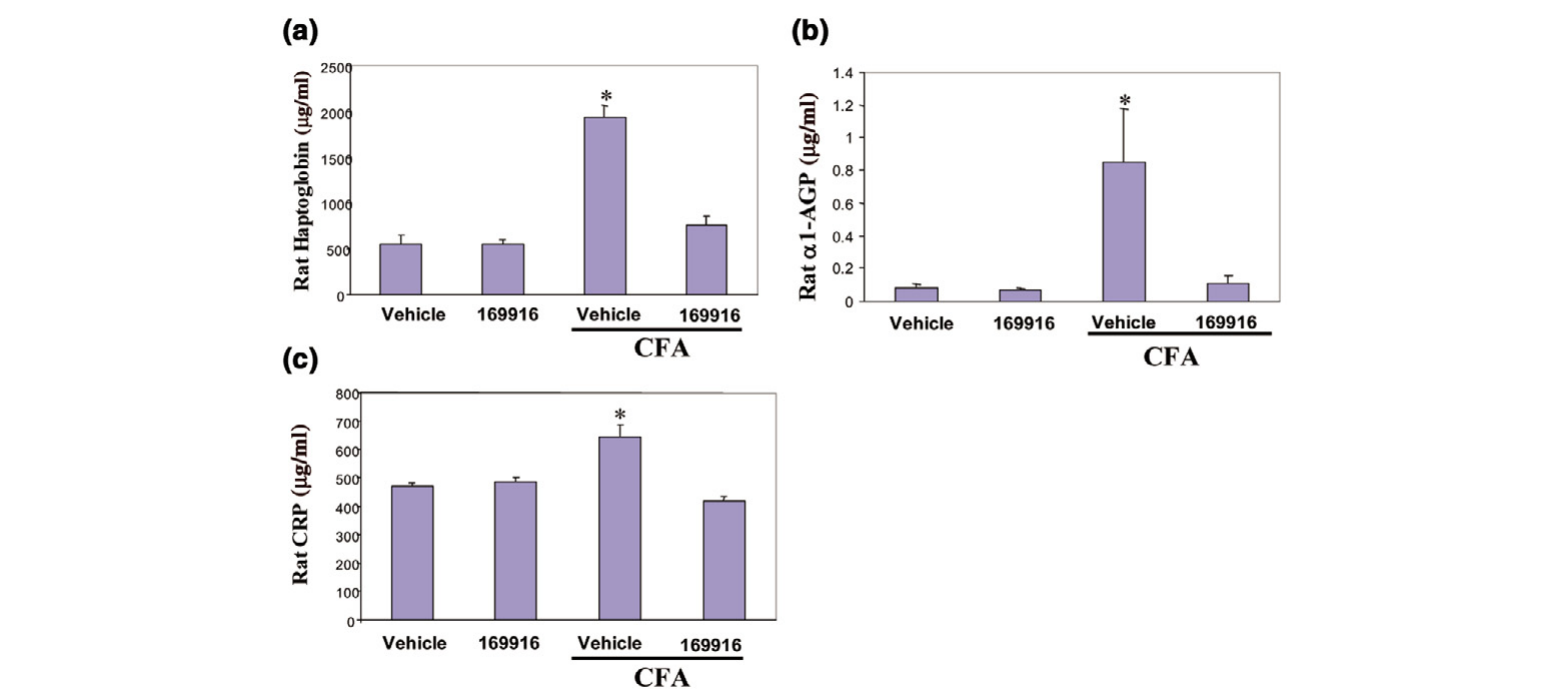
WAY-169916 inhibits the adjuvant-induced expression of serum acute-phase protein. Serum from control male rats or adjuvant-induced rats treated with either vehicle or WAY-169916 (5 mg/kg) was analyzed for the expression of **(a) **haptoglobin, **(b) **α1-acid glycoprotein (α1-AGP) or **(c) **C-reactive protein (CRP) by radial immunodiffusion assay. Results are expressed as means ± SEM from six rats per group. *P < 0.05 compared with vehicle control. CFA, complete Freund's adjuvant.

Because WAY-169916 has been shown to antagonize NF-κB transcriptional activity selectively [27], we wished to begin to address how WAY-169916 might be functioning to improve disease symptoms in the rat adjuvant model. Previous studies have shown changes in concentrations of rat serum proteins induced by adjuvant administration (reviewed in [30]). We decided to look at three acute-phase proteins, haptoglobin, α1-AGP, and CRP, that are induced by the adjuvant and have been correlated with RA progression in humans [31]. Serum was analyzed from male Lewis rats treated with 5 mg/kg WAY-169916 for 10 days. As shown in Fig. 3, both haptoglobin and α1-AGP serum levels were induced about 300 to 400% by adjuvant treatment, whereas CRP inductions were more modest (40%); this was consistent with previous reports [30]. WAY-169916 inhibited the adjuvant induction of all three acute-phase proteins but had no effect on their basal levels.

**Figure 4:**
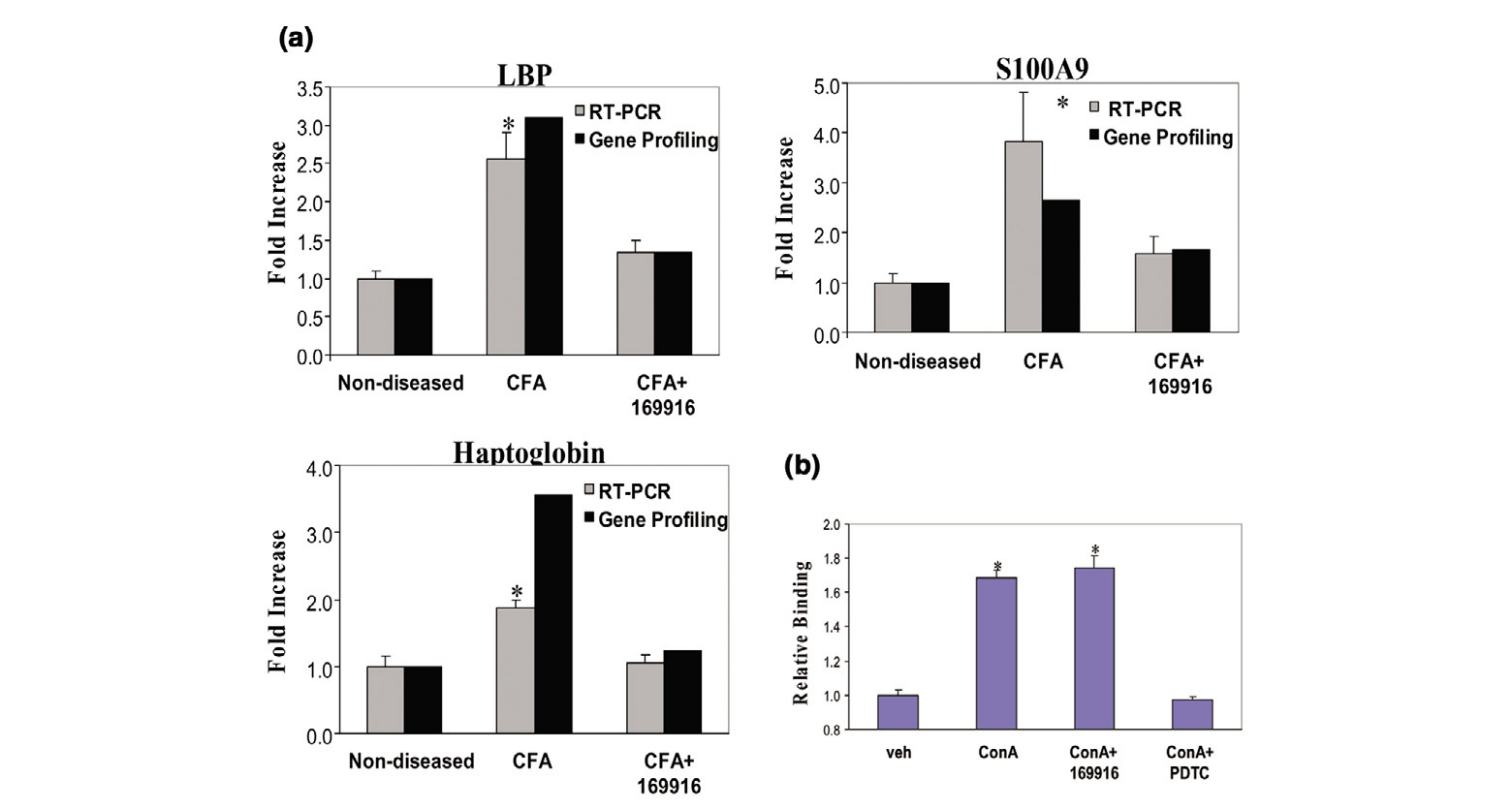
The effect of WAY-169916 in spleen cells. **(a) **The regulation of LBS binding protein (LBP), haptoglobin and S100A9 gene expression by WAY-169916 from spleens from the rat adjuvant model were confirmed by real-time RT–PCR (grey bars) compared with the regulation observed in the gene-profiling experiments (black bars). Results are expressed as means ± SEM from six rats per group. *P < 0.05 compared with vehicle control. **(b) **Treatment with WAY-169916 does not interfere with the binding of NF-κB to DNA. Nuclear extracts from primary mouse spleen cell cultures were co-treated for 18 hours with vehicle or concanavalin A (ConA; 5 μg/ml) and either WAY-169916 (1 μM) or pyrrolidine dithiocarbamate (PDTC) (100 μM) as indicated. CFA, complete Freund's adjuvant.

We also performed gene expression profile analysis from the spleen, liver, and popliteal lymph nodes from these rats. In the spleen, 36 genes were identified that were induced twofold by adjuvant treatment (average fold change; Table 3). Of those 36 genes, WAY-169916 decreased the expression of 29 of them by at least 50%. Several genes that have been implicated in the pathogenesis of RA that were regulated by WAY-169916 include LBS binding protein (LBP), CD14, MMP-9, IL1R2, S100A8, and S100A9. As a control, the regulation of LBP, haptoglobin, and S100A9 was confirmed by real-time RT–PCR (Fig. 4a). A similar global inhibition of adjuvant-induced genes by WAY-169916 was also observed in liver and popliteal lymph node gene-profiling studies. In the liver, 47 genes were induced and WAY-169916 inhibited 43 of those by 50%; in the lymph node, 143 genes were induced and 61 of those were repressed by 50% by WAY-169916 (data not shown).

In addition, we attempted to determine whether treatment with WAY-169916 resulted in direct interference of NF-κB DNA binding in primary spleen cell cultures. The cells were stimulated with ConA (5 μg/ml) for 24 hours and co-treated with either WAY-169916 (1 μM) or PDTC (100 μM), a general inhibitor of NF-κB. As shown in Fig. 4b, activation by ConA resulted in an 80% increase in NF-κB DNA binding. Although PDTC treatment could completely block NF-κB activation, WAY-169916 was without effect. Control experiments demonstrated that the binding of NF-κB was specific, because competition experiments with wild-type oligonucleotide interfered with binding activity whereas a mutated oligonucleotide was without effect (data not shown). These results are consistent with our previous observations [16,27] demonstrating that liganded ER inhibits NF-κB at the transcriptional level downstream from NF-κB DNA binding. Overall, these data indicate a marked anti-inflammatory effect for WAY-169916 that seems to cross multiple signaling pathways and tissues consistent with NF-κB's ubiquitous role in inflammation.

### WAY-169916 anti-inflammatory activity in synoviocytes isolated from patients with RA

**Figure 5:**
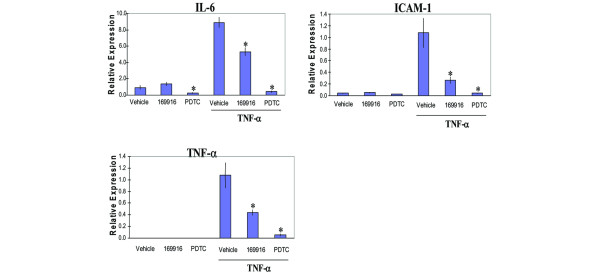
Inhibitory effect of treatment with WAY-169916 on inflammatory gene expression in FLS induced by TNF-α. Fibroblast-like synoviocytes (FLS) were pretreated for 1 hour with WAY-169916 (1 μM) or pyrrolidine dithiocarbamate (PDTC) (100 μM) before treatment with tumor necrosis factor-α (TNF-α) for 1 hour. The mRNA levels for TNF-α, IL-6 and intercellular cell-adhesion molecule-1 (ICAM-1) were determined by real-time RT–PCR and normalized to glyceraldehyde-3-phosphate dehydrogenase. Results are reported as means ± SEM for each group, with the mean level of the stimulated cells treated with vehicle defined as 1. *P < 0.05 compared with vehicle control.

Finally, we wished to test whether WAY-169916 is active in FLS, a human cell type that is thought to have a pathologic function in joint destruction through its production of inflammatory cytokines and MMPs [32]. Activation of NF-κB in FLS is necessary for the production of these inflammatory mediators [5,6]. FLS obtained from male patients with RA were stimulated with TNF-α and treated with vehicle, WAY-169916 (1 μM), or PDTC (100 μM). RNA was analyzed for gene expression changes of intercellular cell-adhesion molecule-1 (ICAM-1), IL-6, and TNF-α by real-time RT–PCR. As shown in Fig. 5, TNF-α-stimulated expression of all three inflammatory genes was significantly blocked by both WAY-169916 and PDTC, which was consistent with previous observations [5]. The cells were confirmed to express ERα mRNA [33] but no ERβ mRNA was detected (data not shown). In total, these data suggest the potential utility of non-steroidal selective NF-κB modulators such as WAY-169916 in treating patients with RA.

## Discussion

RA might occur as a result of an autoimmune response, and recent studies suggest that hypersensitivity to microbial antigens contributes to the development of the arthritis. Microbial or self-antigen presentation to T lymphocytes results in chronic activation of the immune system. Multiple proinflammatory mediators, including IL-1, TNF-α, interferon-γ, and MMPs mediate the inflammation of the joints. Biochemical and histological changes in synovial tissue, cartilage, and bone have been documented in various animal models of arthritis [34,35]. In many respects the synovial and cartilage lesions that develop in these models closely resemble those seen in rheumatoid arthritis. We have investigated the role of WAY-169916 in two such models.

The HLA-B27 transgenic rats spontaneously develop arthritis similar to the human spondyloarthropathies associated with the HLA-B27 and β_2_-microglobulin genes through a T cell-mediated process [34]. In this model, WAY-169916 restored the clinical joint scores to baseline after 10 days. Histological scoring of synovitis and cartilage damage in the tarsal joints after 29 days of treatment was also significantly improved with WAY-169916 treatment.

In the rat adjuvant-induced arthritis model [36], 3 to 6 days after the injection of adjuvant, induction of an αβ T cell response occurs and leads to clinical lesion development in the tarsal joints within 5 to 8 days. Because activated NF-κB was detected in the synovial lining layer and around blood vessels in the inflamed synovium as early as day 3 after adjuvant injection in the Lewis rats and is thought to be correlated with disease development [37], this model was used to test the therapeutic treatment with WAY-169916. We demonstrated that WAY-169916 was effective in improving both joint and histology scores at doses as low as 0.3 mg/kg given orally once daily. Improvement in the synovitis and Mankin scores did occur with higher doses of WAY-169916 even though the joint score reduction was already maximal at 0.3 mg/kg. When the rats were dosed at 5 mg/kg the total synovitis score decreased to 4.14 (data not shown). The beneficial effects of WAY-169916 on joint histology might therefore continue with increasing dose or longer exposure.

A benefit on arthritis progression with non-selective estrogens such a 17β-estradiol has also been demonstrated in both the rat adjuvant-induced arthritis model [38] and the collagen-induced mouse model [39,40]. Indeed, 17β-estradiol has been shown to affect several processes involved in the pathogenesis of RA, including immunoregulation, regulation of adhesion molecules, and modulation of cytokine signaling. However, the role of estrogen has not been well defined in patients with RA. There is evidence that gender might affect the occurrence and progression of RA. Women have a higher risk of developing RA than men. During pregnancy, the disease activity is ameliorated in 75% of women, whereas after delivery, flares occur in up to 90% of patients [41]. The highest incidence of developing RA coincides with menopause, indicating that a decrease in estrogen production might increase the risk of joint inflammation. In a recent randomized clinical trial, post-menopausal women taking hormone therapy had suppressed signs of inflammation and significantly improved disease severity scores (DS28) after 12 months of treatment, which was consistent with previous trials [42].

With the identification of selective NF-κB transcriptional inhibitors such as WAY-169916, the expectation is to accentuate the anti-inflammatory, anti-rheumatic activity observed with the non-selective estrogens. Whereas non-selective estrogens have been documented to contain an anti-inflammatory activity through the suppression of NF-κB transcriptional activity [11,12], hormone therapy can simultaneously elicit both proinflammatory and anti-inflammatory activities as exemplified by the decrease in haptoglobin and α1-AGP levels in women taking hormone therapy [43] while also inducing MMP9 and CRP levels [44,45]. In the rat adjuvant model, WAY-169916 inhibited the adjuvant induction of CRP levels and those of haptoglobin and α1-AGP. Moreover, WAY-169916 had no effect on the basal levels of CRP whereas treatment with estradiol has been shown to increase rat CRP serum levels [46], suggesting a potential differential effect of WAY-169916 in comparison with estradiol. This differential activity has been demonstrated on several classic estrogenic effects. WAY-169916 neither stimulates creatine kinase gene expression driven via an estrogen receptor response element *in vitro *nor promotes uterine proliferation *in vivo *[27] while retaining the anti-inflammatory activity as demonstrated here.

The anti-inflammatory activity of WAY-169916 was further demonstrated in a series of gene-profiling experiments. In the spleens from the adjuvant-treated rats, 36 genes were identified that were induced greater than twofold by the adjuvant treatment. WAY-169916, when dosed at 5 mg/kg, repressed 29 of those genes by at least 50%, and 17 of them by more than 75%. An attractive hypothesis for WAY-169916-mediated activity in the spleen involves the downregulation of LBP and CD14 expression on monocytes and macrophages, resulting in a diminished immune response and ultimately resulting in the observed decreases in MMP9, IL1R2, chemokine-like factor 1, S100A8, and S100A9 through the repression of NF-κB activity [47,48]. In spleen cell cultures, WAY-169916 treatment did not interfere with ConA-stimulated NF-κB DNA binding; however, the downregulation of S100A9 mRNA was confirmed (data not shown), which was consistent with our hypothesis that ER regulates NF-κB at the transcriptional level [16,27]. A similar suppression of adjuvant-induced inflammatory gene expression was also observed in liver and lymph node studies. These data demonstrate that WAY-169916 can have an effect on a global level, both in terms of the tissues targeted and the different inflammatory signaling pathways, to suppress adjuvant-induced gene expression.

Infiltration of inflammatory cells into the synovial tissue and lining layer results in the formation of pannus, a highly vascularized tissue comprising FLS, macrophages, and lymphocytes. FLS are known for their role in joint destruction through the production of cytokines and MMP, which contribute to cartilage degradation (reviewed in [49]). Expression of ER has been detected in synovial tissues from patients with RA [50] and localized to synoviocytes in the synovial lining [22], providing another potential cell type by which WAY-169916 functions. Synoviocytes isolated from a male RA patient were confirmed to express ERα mRNA [33], but no ERβ mRNA was detected (data not shown). The ERα was functional in these cells, because WAY-169916 could effectively block the TNF-α-mediated inflammatory gene expression of IL-6, TNF-α, and ICAM-1. The potential involvement of NF-κB in mediating TNF-α gene induction was demonstrated with the use of a general NF-κB inhibitor, as shown previously [5]. Given the importance of TNF-α signaling in RA disease progression, the ability of WAY-169916 to interfere with this signaling pathway in human synoviocytes suggests a potential clinical benefit for WAY-169916 in patients with RA.

## Conclusions

We detailed the activity of the first pathway-selective ER ligand, WAY-169916, in two models of RA. This compound selectively inhibits NF-κB activity via the ER and imparts significant efficacy in the HLA-B27 and Lewis rat adjuvant-induced models of arthritis. More importantly, no evidence for classic estrogenic activity has been observed with this compound [27]. These data provide evidence that the non-steroidal, pathway-selective ER ligand, WAY-169916, and other compounds in its class might be therapeutically useful in the treatment of RA.

## Abbreviations

α1-AGP = α1-acid glycoprotein; ANOVA = analysis of variance; CFA = complete Freund's adjuvant; ConA = concanavalin A; CRP = C-reactive protein; ER = estrogen receptor; FLS = fibroblast-like synoviocytes; ICAM-1 = intercellular cell-adhesion molecule-1; IκB = inhibitory protein-κB; IL = interleukin; LBP = LBS binding protein; MMP = matrix metalloproteinase; NF-κB = nuclear factor-κB; PDTC = pyrrolidine dithiocarbamate; RA = rheumatoid arthritis; RT–PCR = reverse transcriptase polymerase chain reaction; TNF-α = tumor necrosis factor-α.

## Competing interests

The authors are employees of Wyeth.

## Authors' contributions

JCK, LMA and YL performed the *in vivo *experiments. MF and LW performed the gene-profiling experiments. LBM performed the cell-based assays and serum analysis. CCC, RJS and DCH were involved in the conception and identification of the molecule, and DCH wrote the manuscript. All authors contributed intellectually to the work and read and approved the final manuscript.

## Figures and Tables

**Table 1:** Histological scoring of synovitis and cartilage damage in the tarsal joints from HLA-B27 transgenic rats

Group	Synovial structure (0–3)	Fibroplasia (0–3)	Inflammatory cells (0–3)	Pannus (0–2)	Total synovitis score (0–11)
T/MC vehicle	3.00 ± 0.00	2.80 ± 0.45	3.00 ± 0.00	1.60 ± 0.89	10.40 ± 1.34
WAY-169916, 10 mg/kg	1.80 ± 0.45*	1.20 ± 0.84*	1.40 ± 0.55*^†^	0.40 ± 0.89	5.00 ± 2.24*^†^
Prednisolone, 0.6 mg/kg	2.40 ± 0.55	2.00 ± 0.71*	2.00 ± 0.00*	1.60 ± 0.89	8.00 ± 1.87

Group	Cartilage structure (0–6)	Cartilage cells	Safranin-O/Fast Green staining (0–4)	Tidemark integrity (0–2)	Total Mankin score

T/MC vehicle	3.80 ± 0.84	2.80 ± 0.45	3.20 ± 0.45	0	9.80 ± 1.48
WAY-169916, 10 mg/kg	2.20 ± 0.45*^†^	2.00 ± 0.45*^†^	1.60 ± 0.55*^†^	0	5.80 ± 0.84*^†^
Prednisolone, 0.6 mg/kg	3.40 ± 0.55	2.20 ± 0.45*	3.00 ± 0.00	0	8.60 ± 0.89

Results are means ± SD.

*Significantly less than vehicle (*P *< 0.005). ^†^Significantly less than prednisolone (*P *< 0.005).

**Table 2:** Histological scoring of synovitis and cartilage changes in the tarsal joints from rats with adjuvant-induced arthritis

Group	Synovial structure (0–3)	Fibroplasia (0–3)	Inflammatory cells (0–3)	Pannus (0–2)	Total synovitis score (0–11)
T/MC vehicle	2.92 ± 0.21	2.67 ± 0.41	2.92 ± 0.21	2.00 ± 0.00	10.5 ± 0.63
WAY-169916, 0.3 mg/kg	2.33 ± 0.41*	2.33 ± 0.52	1.58 ± 0.38*	1.17 ± 0.75	7.42 ± 1.88*
WAY-169916, 0.1 mg/kg	2.17 ± 0.68*	1.92 ± 0.49*	1.50 ± 0.45*	0.83 ± 0.98*	6.42 ± 2.90*

Group	Cartilage structure (0–6)	Cartilage cells (0–3)	Safranin-O/Fast Green staining (0–4)	Tidemark integrity (0–1)	Total Mankin score (0–14)

T/MC vehicle	3.52 ± 0.42	2.33 ± 0.41	3.00 ± 0.00	0	8.58 ± 0.74
WAY-169916, 0.3 mg/kg	1.75 ± 0.69*	1.58 ± 0.38*	1.83 ± 0.41*	0	5.17 ± 1.77*
WAY-169916, 0.1 mg/kg	2.25 ± 0.42*	1.42 ± 0.49*	1.67 ± 0.41*	0	5.33 ± 1.21*

Results are means ± SD.

*Significantly less than vehicle (*P *< 0.005).

**Table 3:** WAY-169916 gene-profiling experiment with spleen from rat adjuvant arthritis model

	Av. control	Av. CFA	Av. CFA + WAY-169916	AFC CFA	AFC WAY-169916	Inhibition by WAY-169916 (%)
Transcription factors						
CCAAT/enhancer binding protein (C/EBP), beta	16.8	39.8	19.6	2.37	0.49	87.6
NF-E2-related factor 2	14.2	30.3	19.6	2.13	0.65	66.2
Immune mediators						
Mast cell protease 2	5.3	11.8	5.9	2.20	0.50	91.4
Arachidonate 5-lipoxygenase-activating protein	18.9	46.5	23.9	2.46	0.51	82.0
Chemokine-like factor 1	10.5	28.9	18.9	2.75	0.65	54.3
Phospholipase A_2_, group IIA	33.8	71.7	60.3	2.12	0.84	30.0
Proteoglycan 2, bone marrow	27.6	147.4	59.1	5.34	0.40	73.7
Immune related						
CD14 antigen	15.9	32.3	19.1	2.04	0.59	80.2
Peptidoglycan recognition protein	10.2	39.6	14.5	3.88	0.37	85.2
Lipopolysaccharide-binding protein	3.3	10.2	4.4	3.10	0.43	83.6
IL-1 receptor, type II	2.2	8.1	3.5	3.71	0.44	76.8
Defensin RatNP-3 precursor	31.7	105.3	63.5	3.32	0.60	56.8
Suppressor of cytokine signalling 3	15.2	35.3	15.2	2.33	0.43	99.7
Complement component 3	13.3	28.4	24.2	2.14	0.85	27.6
Defensin NP-2 precursor	58.9	166.6	102.8	2.83	0.62	59.3
Defensin NP-4 precursor	54.1	164.9	104.7	3.05	0.64	54.3
Paired immunoglobulin-like receptor-B	11.0	22.7	18.3	2.07	0.81	37.6
25 oligoadenylate synthetase	24.6	51.4	24.8	2.09	0.48	99.2
Tumor necrosis factor receptor II	7.3	15.8	8.9	2.18	0.56	81.0
S100 calcium-binding protein A8 (calgranulin A)	52.2	184.5	91.5	3.54	0.50	70.3
S100 calcium-binding protein A9 (calgranulin B)	144.9	382.7	242.1	2.64	0.63	59.1
Ficolin B	42.3	121.1	74.7	2.86	0.62	58.9
Myelin and lymphocyte protein	11.8	27.6	19.9	2.34	0.72	49.1
Protease						
Matrix metalloproteinase 9	2.0	8.4	3.5	4.24	0.42	75.8
Chymase 1	2.9	9.2	5.1	3.16	0.56	64.8
Transport						
Monocarboxylate transporter	10.3	24.4	10.0	2.38	0.41	102.3
Lipocalin 2	16.3	55.3	33.0	3.39	0.60	57.1
Acute phase						
Haptoglobin	10.5	37.5	12.9	3.58	0.34	90.9
Metabolism						
Uridine phosphorylase I	8.9	32.8	9.1	3.68	0.28	99.3
Guanine deaminase	17.8	39.5	20.7	2.21	0.52	86.9
Microsomal glutathione S-transferase 1	40.0	87.6	51.2	2.19	0.58	76.6
GTP cyclohydrolase 1	11.6	25.5	23.6	2.20	0.93	13.8
Hepatic steroid hydroxylase II A_2_	9.0	18.7	21.5	2.07	1.15	-29.0
Adhesion						
C-CAM4 protein	11.5	24.5	16.0	2.12	0.65	65.6
Fibronectin 1	57.2	116.6	91.3	2.04	0.78	42.6
Unknown function						
Expressed sequence tag	20.0	41.5	25.1	2.08	0.61	76.1

AFC, average fold change; Av., average; CFA, complete Freund's adjuvant.
